# Dual blockade of EGFR and PI3K signaling pathways offers a therapeutic strategy for glioblastoma

**DOI:** 10.1186/s12964-023-01400-0

**Published:** 2023-12-18

**Authors:** Tongxuan Guo, Changyong Wu, Junhao Zhang, Jiefeng Yu, Guoxi Li, Hongyan Jiang, Xu Zhang, Rutong Yu, Xuejiao Liu

**Affiliations:** 1 Insititute of Nervous System Diseases, Xuzhou Medical University, Xuzhou, Jiangsu China; 2 Department of Neurosurgery, Affiliated Hospital of Xuzhou Medical University, Xuzhou, Jiangsu China

**Keywords:** Glioblastoma, AZD-9291, GDC-0084, Combination therapy, Synergistic inhibition

## Abstract

**Background:**

Glioblastoma multiforme (GBM) is a devastating disease that lacks effective drugs for targeted therapy. Previously, we found that the third-generation epidermal growth factor receptor (EGFR) inhibitor AZD-9291 persistently blocked the activation of the ERK pathway but had no inhibitory effect on the phosphoinositide 3-kinase (PI3K)/Akt pathway. Given that the PI3K inhibitor GDC-0084 is being evaluated in phase I/II clinical trials of GBM treatment, we hypothesized that combined inhibition of the EGFR/ERK and PI3K/Akt pathways may have a synergistic effect in the treatment of GBM.

**Methods:**

The synergistic effects of cotreatment with AZD-9291 and GDC-0084 were validated using cell viability assays in GBM and primary GBM cell lines. Moreover, the underlying inhibitory mechanisms were assessed through colony formation, EdU proliferation, and cell cycle assays, as well as RNA-seq analyses and western blot. The therapeutic effects of the drug combination on tumor growth and survival were investigated in mice bearing tumors using subcutaneously or intracranially injected LN229 xenografts.

**Results:**

Combined treatment with AZD-9291 and GDC-0084 synergistically inhibited the proliferation and clonogenic survival, as well as induced cell cycle arrest of GBM cells and primary GBM cells, compared to monotherapy. Moreover, AZD-9291 plus GDC-0084 combination therapy significantly inhibited the growth of subcutaneous tumors and orthotopic brain tumor xenografts, thus prolonging the survival of tumor-bearing mice. More importantly, the combination of AZD-9291 and GDC-0084 simultaneously blocked the activation of the EGFR/MEK/ERK and PI3K/AKT/mTOR signaling pathways, thereby exerting significant antitumor activity.

**Conclusion:**

Our findings demonstrate that the combined blockade of the EGFR/MEK/ERK and PI3K/AKT/mTOR pathways is more effective against GBM than inhibition of each pathway alone, both in vitro and in vivo. Our results suggest that AZD-9291 combined with GDC-0084 may be considered as a potential treatment strategy in future clinical trials.

Video Abstract

**Supplementary Information:**

The online version contains supplementary material available at 10.1186/s12964-023-01400-0.

## Introduction

Glioblastoma multiforme (GBM) is the most common malignant brain tumor in adults and is characterized by a short disease course, high recurrence rate, and high mortality rate. Although GBM has a lower incidence than most other systemic tumors, it grows rapidly and invades important life-regulating centers around the tumor [1]. In addition, regardless of advancements in the multimodal treatment for GBM, the average 5-year survival rate of patients has not improved significantly [2]. Temozolomide has significant blood-brain barrier permeability and is the first-line chemotherapy drug against GBM. Although temozolomide can prolong the survival and improve the quality of life of patients to a certain extent, its use still has several disadvantages, such as side effects and drug resistance [3]. Hence, it is essential to development new therapeutic drugs and more effective treatment strategies.

Epidermal growth factor receptor (EGFR) is a tyrosine kinase receptor, which is encoded by the proto-oncogene c-ErbB1 and is one of the four members of the HER/ErbB family [4]. EGFR amplification is the most common genetic alteration in GBM [5]. EGFR activation is closely related to the growth and invasion of GBM cells and is an effective target for anti-GBM therapy [6, 7]. Although the first-generation EGFR inhibitors, gefitinib and erlotinib, and the second-generation EGFR inhibitor afatinib have been shown to inhibit GBM cell growth, proliferation, and angiogenesis in preclinical studies, these EGFR inhibitors have not shown therapeutic efficacies in clinical trials [8, 9]. This discrepancy may be due to several possible reasons. First, the tested agents (mainly erlotinib, gefitinib, and lapatinib) have poor brain penetration. Second, it is possible that the EGFR inhibitor (such as gefitinib) blocks EGFR but not the downstream proteins involved in EGFR signal transduction [10–12]. AZD-9291 is an oral, third-generation tyrosine kinase inhibitor that irreversibly inhibits EGFR. AZD-9291 has significant brain penetration, resulting in 5–25-fold higher levels in brain tissue compared to plasma [13, 14]. Our previous research demonstrated that AZD-9291 effectively inhibits the growth of GBM cells in vitro and in vivo [15]*.* Furthermore, it prolongs the survival of GBM-bearing mice. Compared to gefitinib and erlotinib, AZD-9291 has approximately a 10-fold higher inhibitory activity on GBM cells. Importantly, we found that AZD-9291 persistently blocked the activation of the EGFR/ERK pathway to overcome primary resistance [15]. Recently, the clinical activity of AZD-9291 in human GBM was reported. The results showed that half of the patients achieved either partial response or stable disease [16]. Because of the extreme complexity of the abnormal signaling pathways in GBM, inhibition of a single pathway may not achieve the desired therapeutic effect. Several studies have shown that targeting the phosphoinositide 3-kinase (PI3K)/Akt/mammalian target of rapamycin (mTOR) pathway increases the antitumor activity of EGFR inhibition [17, 18].

The PI3K pathway is a key signaling pathway in tumorigenesis and tumor progression, mediating a variety of tumor cell functions, including growth, migration, invasion, angiogenesis, and metabolism [19]. Mutations, amplification, and deletion of key signaling proteins of the PI3K pathway, such as EGFR, PTEN, and PI3K, were detected in more than 80% of GBM cases [20]. Thus, the PI3K pathway is an ideal target for the treatment of GBM. GDC-0084 is a potent, oral, and selective dual PI3K/mTOR-inhibitor that was developed in recent years [21]. It exhibits significant tumor growth inhibition in preclinical models of glioblastoma [22]. A clinical phase I study of GDC-0084 was conducted in patients with progressive or recurrent high-grade glioma (NCT01547546) [23]. The results showed that GDC-0084 had a favorable safety profile. Importantly, the ratio of GDC-0084 concentration in the brain to that in the plasma was approximately 1.0, indicating that GDC-0084 had significant brain penetration [23]. An ongoing phase II clinical trial of GDC-0084 is exploring the safety, tolerability, and clinical activity of GDC-0084 in newly diagnosed GBM with unmethylated MGMT promoter status (NCT03522298) [24]. The results demonstrated that, for response-evaluable patients, the median progression-free survival was 8.4 months, and 25% of patients remained free of progression after 15 months of follow-up [24]. The existing clinical trial results further support the potential advantages of GDC-0084 for the treatment of GBM.

Because the EGFR inhibitor AZD-9291 blocks ERK pathway activation with no significant effect on the Akt pathway, we hypothesized that combined inhibition of the EGFR/EKR and PI3K/AKT pathways may have a synergistic effect in the treatment of GBM. This study investigated the effect of AZD-9291 combined with GDC-0084 on the growth of GBM cells, primary GBM cells, and GBM xenografts, providing a new treatment option for improving the therapeutic effect of GBM and providing a scientific basis for further clinical trials of this combined treatment strategy.

## Materials and methods

### Culture of cell lines

The human GBM cell lines (LN229, U251, U87 and T98G) were purchased from Shanghai Cell bank, Type Culture Collection Committee, Chinese Academy of Sciences. GBM1, GBM2 and GBM3 are primary GBM cells established by our laboratory. All cell lines were cultured in Dulbecco’s modified Eagle’s medium (DMEM) supplemented with 10% fetal bovine serum (FBS) and grown in a humidified incubator at 37 °C with 5% CO_2_.

### Antibodies and inhibitors

Primary antibodies against p-EGFR (#3777), EGFR (#2232), p-c-Raf (#9421), p-MEK1/2 (#9154), ERK1/2 (#4695), p-ERK1/2 (#4376), p-p90RSK (#9341), p-AKT (#4060), AKT (#9272), p-mTOR (#5536), mTOR (#2983), p-p70S6 (#9206), p70S6 (#9202), p-4E-BP1 (#2855), 4E-BP1 (#9644), cyclin D1 (#2922), p21 (#2947), β-actin (#4970) and GAPDH (#97166) were purchased from Cell Signaling Technology (CST, MA, US). MEK1/2 (#ABP0100) antibody was obtained from Abbkine (Wuhan, China). Anti-Ki67 (#PA5–16446) was purchased from Thermo Fisher (Waltham, MA, USA).

EGFR inhibitor AZD-9291 and dual PI3K/mTOR inhibitor GDC-0084 were obtained from CSNpharm (CSNpharm, Chicago, IL, US). These inhibitors were dissolved in DMSO to create a 10 mmol/L solution, which was diluted to different concentrations in DMEM medium before use.

### CCK-8 assay

The cytotoxicity of AZD-9291- and/or GDC-0084-treated GBM cells was measured by CCK-8 assay (Vicmed, Jiangsu, China). Briefly, GBM cells or primary GBM cells (4 × 10^3^/well) were seeded in 96-well plates with 3 replicate wells per group. After overnight incubation, different concentrations of AZD-9291 and/or GDC-0084 were added into each well for 72 hours. CCK-8 was added and cells were incubated for 1 hour. The absorbance was detected at 450-nm wavelength with a microplate reader. The combination index (CI) of AZD-9291 and GDC-0084 was calculated using CompuSyn software (ComboSyn, Inc.).

### Colony formation assay

GBM or primary GBM cells (800 per well) were plated into 6-well plates and treated with AZD-9291 (2 μΜ) and/or GDC-0084 (2 μΜ) for 24 hours, and followed by incubation with drug-free medium and continued culturing for 14 days to allow colony formation. The medium was removed and cells were washed with PBS 3 times, fixed with 4% paraformaldehyde and then were stained with 1% crystal violet. The plates were dried at room temperature and the number of colonies was counted.

### EdU incorporation assay

EdU Cell Proliferation Detection Kit (Abbkine, Hubei, China) was used to evaluate cell proliferation. LN229, U251, T98G, U87, GBM2 and GBM3 cells were seeded in 96-well cell culture plates at 8000 cells/well. Cells were incubated overnight, followed by AZD-9291 and/or GDC-0084 treatment for 24 hours. After incubating with 10 μM EdU for 2 hours, cells were fixed with 4% paraformaldehyde for 30 min. Then, cells were washed with PBS and treated with 0.5% Triton X-100 for 10 min. Last, 100 μL Click-iT was added to each well, and the reaction was incubated for 30 min, followed by DAPI staining for 15 min. All images were captured using the fluorescence microscope (Olympus, Tokyo, Japan).

### RNA-sequencing (RNA-seq) analysis

RNA-seq was performed in four groups of samples, including the control group, AZD-9291 treatment group, GDC-0084 treatment group, and AZD plus GDC (AZD + GDC) treatment group. To improve data accuracy, three replicate samples were tested from each group. All samples were processed by RNA-seq pipeline (Novogene Co., China). The RNA integrity was assessed using a 2100 Bioanalyzer system (Agilent Technologies, Santa Clara, CA) and the RNA Nano 6000 detection kit (Agilent Technologies). The RNA-seq sample library was constructed and sequenced for analysis. After quantitative treatment, differential expression analysis was performed on three biological replicates in each group using the DESeq2 R package. The expression thresholds for significant differences in the analysis was p adj < 0.05 and |log2(foldchange)| > 1. According to the differential expressed genes (DEGs), the Kyoto Encyclopedia of Genes and Genomes (KEGG) and Gene set enrichment analysis (GSEA) were performed using the ggplot2 [3.3.6] R package.

### Cell cycle assay

LN229, U251, U87 or T98G cells were seeded on 6-cm culture dishes, and 2 μM AZD9291 and/or 2 μM GDC-0084 was added after cell adhesion and incubated for 24 hours. Subsequently, the cells were collected for cell cycle analysis and were fixed with 70% ice-cold ethanol overnight, washed twice with PBS, and stained with a solution containing PI/RNase for 15 minutes. Finally, the cell cycle distribution was detected by flow cytometry and analyzed by flow cytometry software.

### Western blot assay

GBM or primary GBM cells were treated with AZD9291 and/or GDC-0084 for 36 hours, and then the total protein was collected for immunoblot analysis as previously described [25]. The protein expression levels of p-EGFR, EGFR, p-c-Raf, p-MEK1/2, MEK1/2, ERK1/2, p-ERK1/2, p-p90RSK, p-AKT, AKT, p-mTOR, mTOR, p-p70S6, p70S6, p-4E-BP1, 4E-BP1, cyclin D1 and p21 were measured by specific antibodies. The expression of β-actin or GAPDH was shown as the loading control.

### Animal experiments

Protocols for animal experiments in this study were approved by the ethics committee of Xuzhou Medical University. We purchased 5–6-week-old male BALB/c athymic nude mice from Weitong Lihua Experimental Animal Technology Co., Ltd. (Beijing, China). We used a subcutaneous tumor model and an orthotopic LN229 xenograft model in mice to evaluate the therapeutic effect of AZD-9291 combined with GDC-0084 on GBM. For the subcutaneous tumor model, LN229 cells (density: 1.5 × 10^6^) were inoculated on the right flank of each nude mouse. Once the tumor grew to a volume of approximately 100–150 mm^3^, the tumor-bearing mice were randomly divided into four groups (*n* = 4 mice per group; 16 mice in total): control group (Vehicle); AZD-9291 treatment group (15 mg/kg of AZD-9291); GDC-0084 treatment group (15 mg/kg of GDC-0084); and AZD + GDC combined treatment group. In each group, animals received a corresponding intervention via an intraperitoneal injection for 4 weeks (5 days of continuous administration, followed by 2 days without administration per week). In each animal, tumor size was measured with calipers every 2 days. The volume of the subcutaneous tumors was calculated according as (length × width^2^)/2 (assuming a prolate shape). The subcutaneous tumor tissues of different groups were marked, numbered, and weighed separately. Cell lysate was added at a weight (g): volume (μL) ratio of 1:10. The tumor tissue was homogenized using a tissue homogenizer and shaken on a shaker at 4 °C for 30 min, followed by centrifugation at 12,000 rpm/min for 10 min. The supernatant was collected for protein quantification and subsequent western blotting to analyze the effects of the combination therapy according to the expression levels of AKT, p-AKT, ERK, and p-ERK1/2 in mice.

For the intracranial tumor model, LN229 cells with stable expression of luciferase were orthotopically inoculated into the right side of the brain of nude mice using a small animal stereotaxic apparatus (1 × 10^6^ cells were injected in each mouse). Three days after tumor cell inoculation, the tumor-bearing mice were randomly divided into four groups (*n* = 9 mice per group): control group (Vehicle); AZD-9291 treatment group (15 mg/kg of AZD-9291); GDC-0084 treatment group (15 mg/kg of GDC-0084); and AZD + GDC combined treatment group. Similar to the subcutaneous tumor model, animals in each group received corresponding interventions via intraperitoneal injections for 4 weeks (5 days of continuous administration, followed by 2 days without administration per week). On Days 7, 14, and 21, intracranial tumor growth was monitored using a small animal in vivo imaging system. On Day 29, three tumor-bearing mice were randomly selected from each group and euthanized. After conventional perfusion, mouse brains were collected and sectioned, followed by hematoxylin and eosin staining to observe the tumor size. The remaining six mice in each group were used for survival analysis. Mice were euthanized at the onset of neurological symptoms caused by tumor progression, such as rotational behavior, decreased activity, and development of a dome-shaped head. Immunohistochemical test was used to detect the effect of AZD-9291 plus GDC-0084 treatment on the expression level of Ki67 in the mice.

### Statistical analysis

The statistical analyses of the experimental data were performed using the statistical software GraphPad Prism 7.0, and the results are presented as mean ± standard deviation. of three independent experiments. The selected chart was one of the results of repeated experiments. Comparisons of the mean values between two groups were performed using Student’s t-test. One-way analysis of variance (ANOVA) was used for the comparison more than two groups. Kaplan-Meier method was used for survival analysis. Log-rank Test was used to compare whether there was a difference in survival time between the two groups. α = 0.05 was determined as the test level. *P < 0.05 was considered as statistical significance in all results.

## Results

### AZD-9291 plus GDC-0084 synergistically inhibits GBM cell proliferation and colony formation

**Fig. 1:**
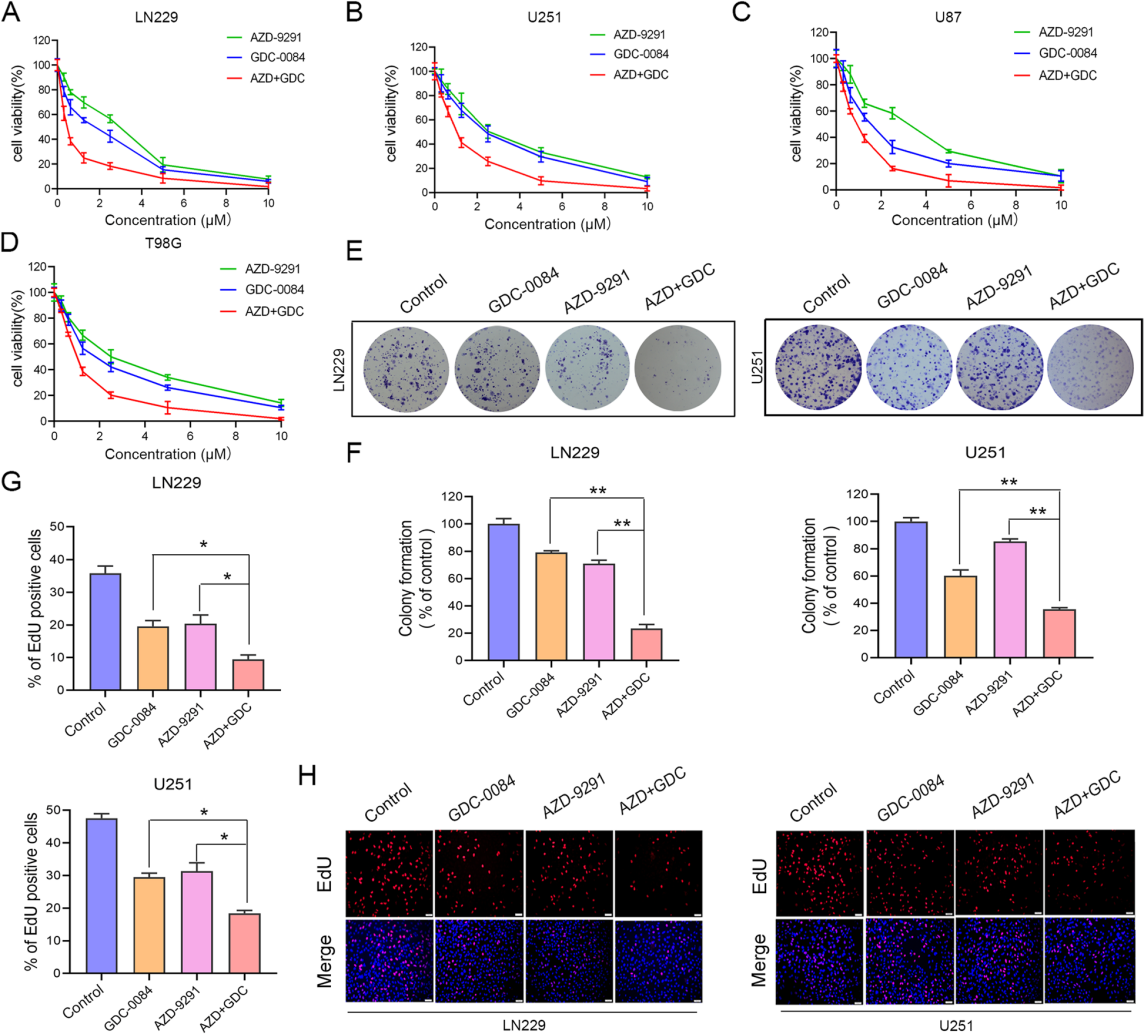
Combinatorial treatment with AZD-9291 and GDC-0084 synergistically inhibits cell viability and colony formation in GBM cells. **A**-**D** Increasing concentrations of AZD-9291, GDC-0084 or both were used to treat four GBM cell lines for 72 h. Cell viability was then measured by CCK-8 assay. **E**, **F** LN229 and U251 cells were treated with AZD-9291 (2 μΜ) and/or GDC-0084 (2 μΜ) for 24 h, and then changed with drug-free medium for another 14 days. The numbers of colony formation were counted. **F** Quantitative analysis of the results in (**E**). The numbers of colony formation were normalized to the control group. **G**, **H** Representative images (**H**) and quantitative results (**G**) of EdU assay in LN229 and U251 cells treated with AZD-9291 (2 μΜ) and/or GDC-0084 (2 μΜ), scale bar: 100 μm. All the data were presented as means ± SD from three independent experiments (*P < 0.05, **P < 0.01)

This study used the cell counting kit-8 (CCK-8, cell viability assay) to evaluate the effects of GDC-0084, AZD-9291, and their combination on the survival of four GBM cell lines. Compared to GDC-0084 or AZD-9291 monotherapy, the combination of GDC-0084 and AZD-9291 inhibited the growth of GBM cells more effectively (Fig. 1A–D). Furthermore, the combination therapy exhibited concentration-dependent effects. The Chou-Talalay method was used to analyze the combined effects of the two inhibitors based on the combination index (CI) of the two drugs. The results showed that the CI of the two inhibitors was < 1, indicating that the two compounds had a synergistic inhibitory effect (Table S1, Supplementary information).

To examine the effect of AZD-9291 combined with GDC-0084 on GBM cell proliferation, the EdU incorporation assay was used to detect cell proliferation. Relative to the control group, the average EdU-positive cell rates of LN229 and U251 under treatment with 2 μΜ AZD-9291 were 20.39 and 31.33%, respectively. After combining AZD-9291 treatment with GDC-0084, the average EdU-positive cell rates of LN229 and U251 were reduced to 9.47 and 18.42%, respectively (Fig. 1G, H). To further analyze the effect of AZD-9291combined with GDC-0084 on the long-term inhibition of GBM cell proliferation, the colony formation assay was used to evaluate the colony formation ability of GBM cells. Compared with the control group, GDC-0084 monotherapy and AZD-9291 monotherapy, AZD-9291 combined with GDC-0084 significantly inhibited the colony formation of GBM cells (Fig. 1E). The colony formation rates of LN229 and U251 cells were reduced to 23.40 and 35.54%, respectively (Fig. 1F). To verify the consistency of the effects of the combination on the proliferation of various GBM cells, U87 and T98G cells were also used for EDU and colony formation assays. The data showed that the combination of AZD-9291 and GDC-0084 more significantly inhibited the proliferation and colony formation ability of U87 and T98G cells (Figs. S1 and S2, Supplementary material). Our results indicated that the combination of AZD-9291 and GDC-0084 synergistically inhibited the proliferation of GBM cells.

### AZD-9291 combined with GDC-0084 promotes cell cycle arrest in G0/G1 phase

**Fig. 2:**
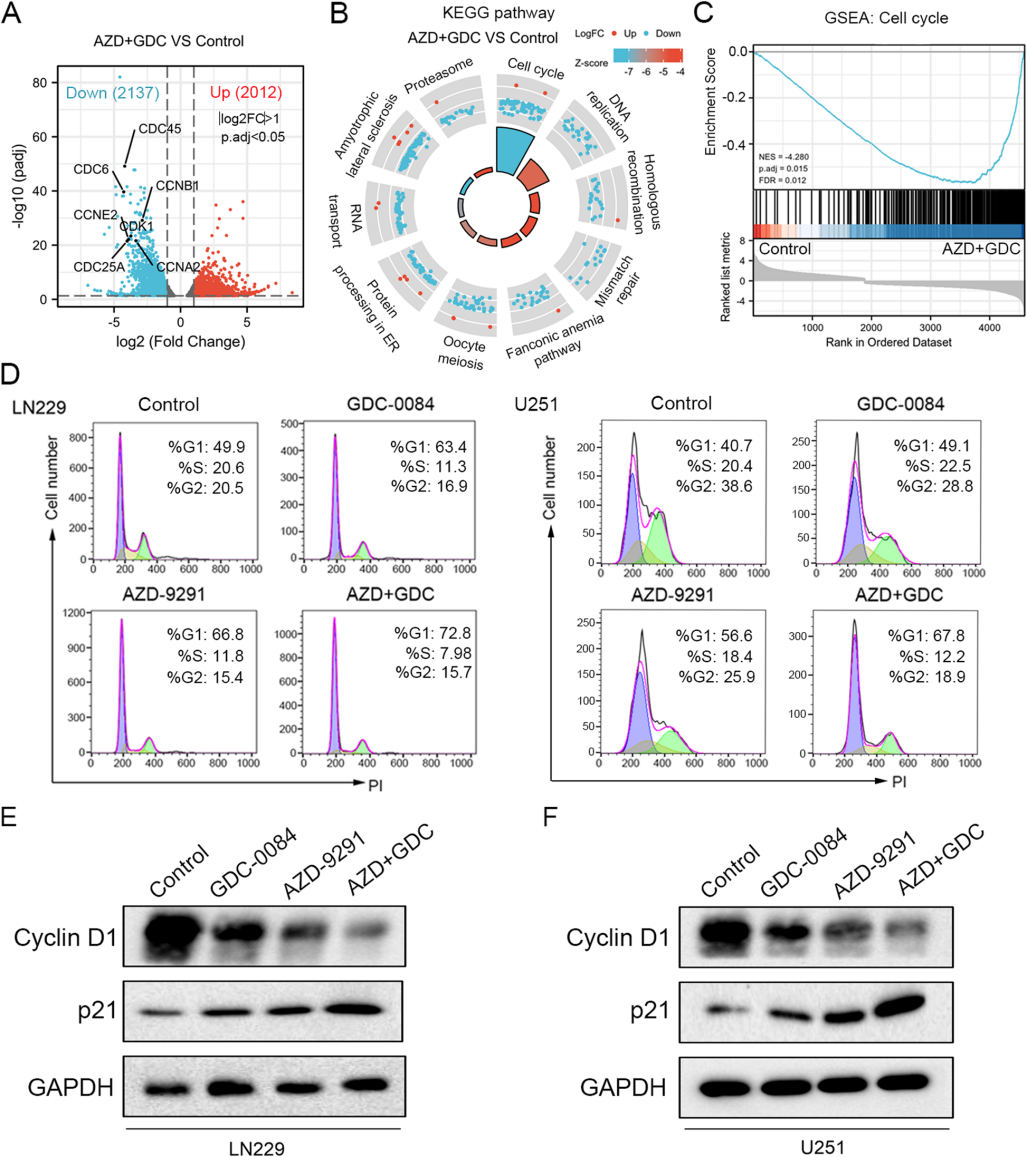
AZD-9291 and GDC-0084 induce cell cycle arrest in G0/G1 phase. **A** The differentially expressed genes (DEGs) were analyzed by transcriptome sequencing after combination treatment. According to the volcano scatter plot of expressed genes, 2021 genes were up-regulated and 2037 genes were down-regulated after AZD-9291 and GDC-0084 combination treatment. |Log2FC| > 1 & p.adj < 0.05. KEGG pathway (**B**) and Gene set enrichment analysis (GSEA) (**C**) enrichment analysis of the DEGs in AZD-9291 and GDC-0084 co-treated cells vs. control cells. **D** Cell cycle analyses of LN229 and U251 treated by AZD-9291 (2 μΜ) and/or GDC-0084 (2 μΜ) by flow cytometry. **E**, **F** LN229 and U251 cells were treated with AZD-9291 (2 μΜ), GDC-0084 (2 μΜ) or combination for 24 h. Cell lysates were analyzed for Cyclin D1 and p21 by western blot analysis

To explore the mechanism by which AZD-9291 combined with GDC-0084 inhibited GBM cell proliferation, high-throughput RNA-seq analysis was performed to screen DEGs and their associated signaling pathways between DMSO, AZD-9291, and/or GDC-0084 treatment groups. As shown in Fig. 2A, comparison between the control and AZD + GDC groups showed 2137 downregulated genes and 2012 upregulated genes. Among the downregulated genes, DEGs related to the regulation of cell cycle progression exhibited the most significant differential expression. In addition, KEGG and GSEA enrichment analyses showed that, compared to the control group, the AZD + GDC group was significantly enriched in cell cycle, DNA replication, and other signaling pathways (Fig. 2B, C). Therefore, we further verified the effect of the drug combination on cell cycle progression using flow cytometry. Compared to the control group, the number of cells in the G0/G1 phase were significantly increased after GDC-0084 or AZD-9291 monotherapy. Nevertheless, the increased number of cells in the G0/G1 phase was more significant after GDC-0084 and AZD-9291 combination therapy (Fig. 2D). Western blotting was performed to detect the expression of G1 phase marker proteins, cyclin D1 and p21, to further evaluate the effect of the combination therapy. Our results showed that, compared to GDC-0084 or AZD-9291 monotherapy, the combination of GDC-0084 and AZD-9291 further decreased the expression level of cyclin D1 and significantly increased the protein expression level of p21 in LN229 and U251 cells (Fig. 2E, F). Meanwhile, the similar results were also observed in U87 and T98G cells (Fig. S3, Supplementary material). In brief, compared to GDC-0084 or AZD-9291 monotherapy, AZD-9291 combined with GDC-0084 was more effective in causing cell cycle arrest in the G0/G1 phase, thereby inhibiting cell proliferation.

### AZD-9291 combined with GDC-0084 simultaneously inhibit the EGFR/ERK and AKT/mTOR signaling pathways

**Fig. 3:**
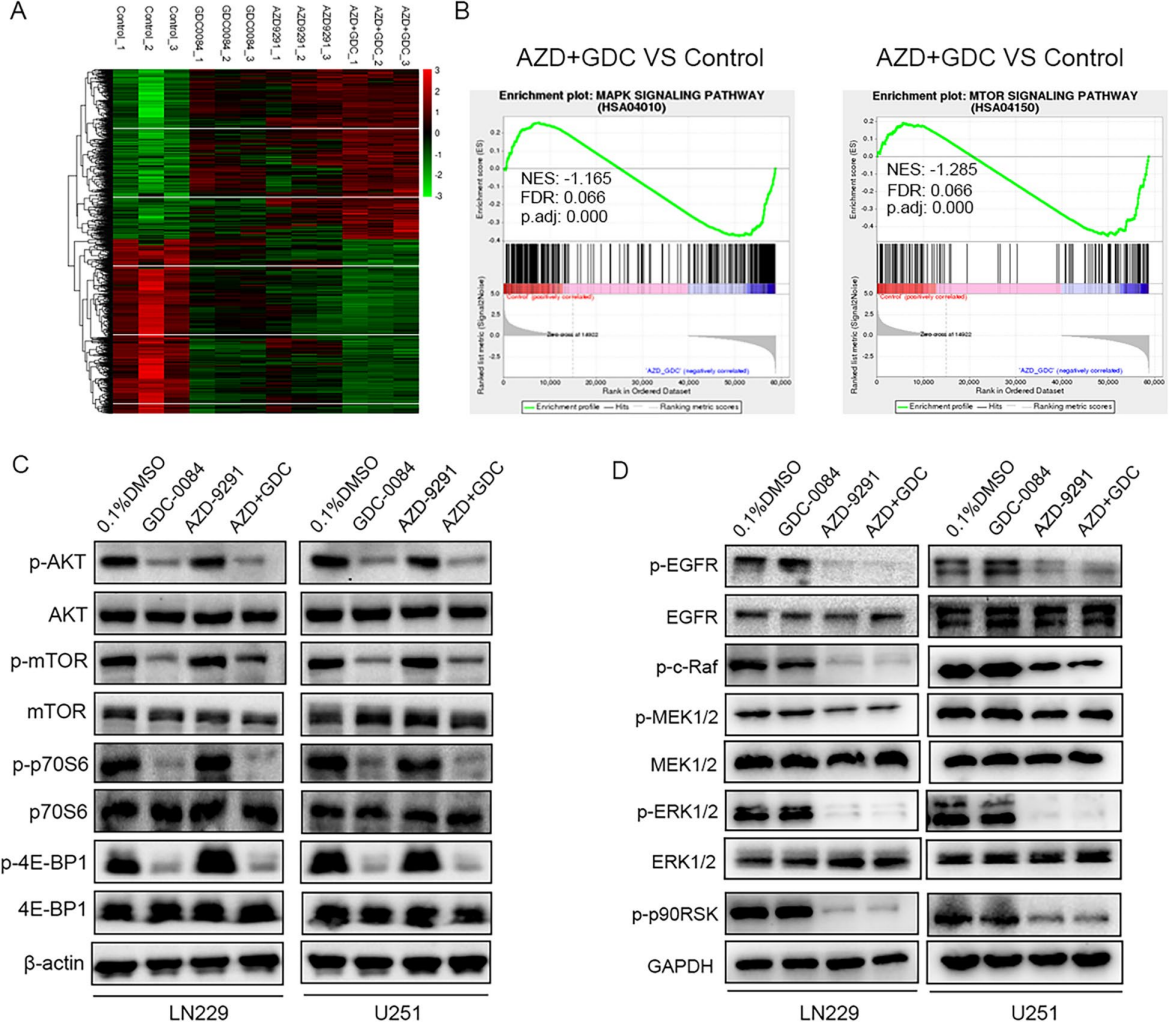
The combination of AZD-9291 and GDC-0084 can simultaneously block EGFR/MEK/ERK and PI3K/AKT/mTOR signaling pathways. **A** DEGs in the control, GDC-0084, AZD-9291 and AZD + GDC treatment groups with triplicates are shown in the heat map. Gradient color barcode indicated fold change of expression (Log2). **B** GSEA was used to analyze the signaling pathways enrichment in different groups. **C**, **D** Representative western blot analysis showing the effects of AZD-9291 combined with GDC-0084 on PI3K/AKT/mTOR and EGFR/MEK/ERK signaling pathways in LN229 and U251 cells. The expression levels of core protein of these two signaling pathways were examined by using indicated antibodies

Our previous study showed that AZD-9291 treatment alone only inhibited the activation of the MEK/ERK signaling pathway downstream of EGFR but had no effect on the AKT/mTOR signaling pathway downstream of EGFR [15]. In the present study, we combined AZD-9291 with GDC-0084 to evaluate their regulatory effect on the downstream MEK/ERK and AKT/mTOR signaling pathways of EGFR and to explore the mechanism underlying the inhibition of GBM growth. This study used heatmaps to visualize the RNA-seq results of the DEGs. Compared with the vehicle, GDC-0084 monotherapy, and AZD-9291 monotherapy, the fold-changes of DEG upregulation and downregulation were more significant after treatment with AZD-9291 and GDC-0084 combination therapy (Fig. 3A). Gene set enrichment analysis showed that DEGs were significantly enriched in the MAPK and mTOR signaling pathways after combination therapy and caused significant suppression of the MAPK and mTOR signaling pathways, which may contribute to the inhibitory effects of AZD + GDC treatment on GBM cell proliferation (Fig. 3B).

We used western blotting to further evaluate the effects of AZD-9291 and/or GDC-0084 treatments on the protein expression levels of important regulatory molecules in the MEK/ERK and AKT/mTOR signaling pathways downstream of EGFR. AZD-9291 monotherapy reduced the phosphorylation levels of EGFR, c-raf, MEK1/2, ERK1/2, and p90RSK in GBM cells, without affecting the phosphorylation levels of AKT, mTOR, p70S6, and 4E-BP1 in the AKT/mTOR signaling pathway, which were consistent with the conclusions of our previous study (Fig. 3C, D). In addition, GDC-0084 monotherapy only reduced the phosphorylation levels of AKT, mTOR, p70S6, and 4E-BP1 in the AKT/mTOR signaling pathway but had no effect on the MEK/ERK signaling pathway in GBM cells (Fig. 3C, D). Interestingly, the combination of GDC-0084 and AZD-9291 simultaneously inhibited the MEK/ERK and the AKT/mTOR signaling pathways (Fig. 3C, D), indicating that this combination inhibited GBM cell proliferation by simultaneously blocking the activation of the MEK/ERK and AKT/mTOR signaling pathways.

### AZD-9291 combined with GDC-0084 synergistically inhibits the proliferation and colony formation of primary GBM cells

**Fig. 4:**
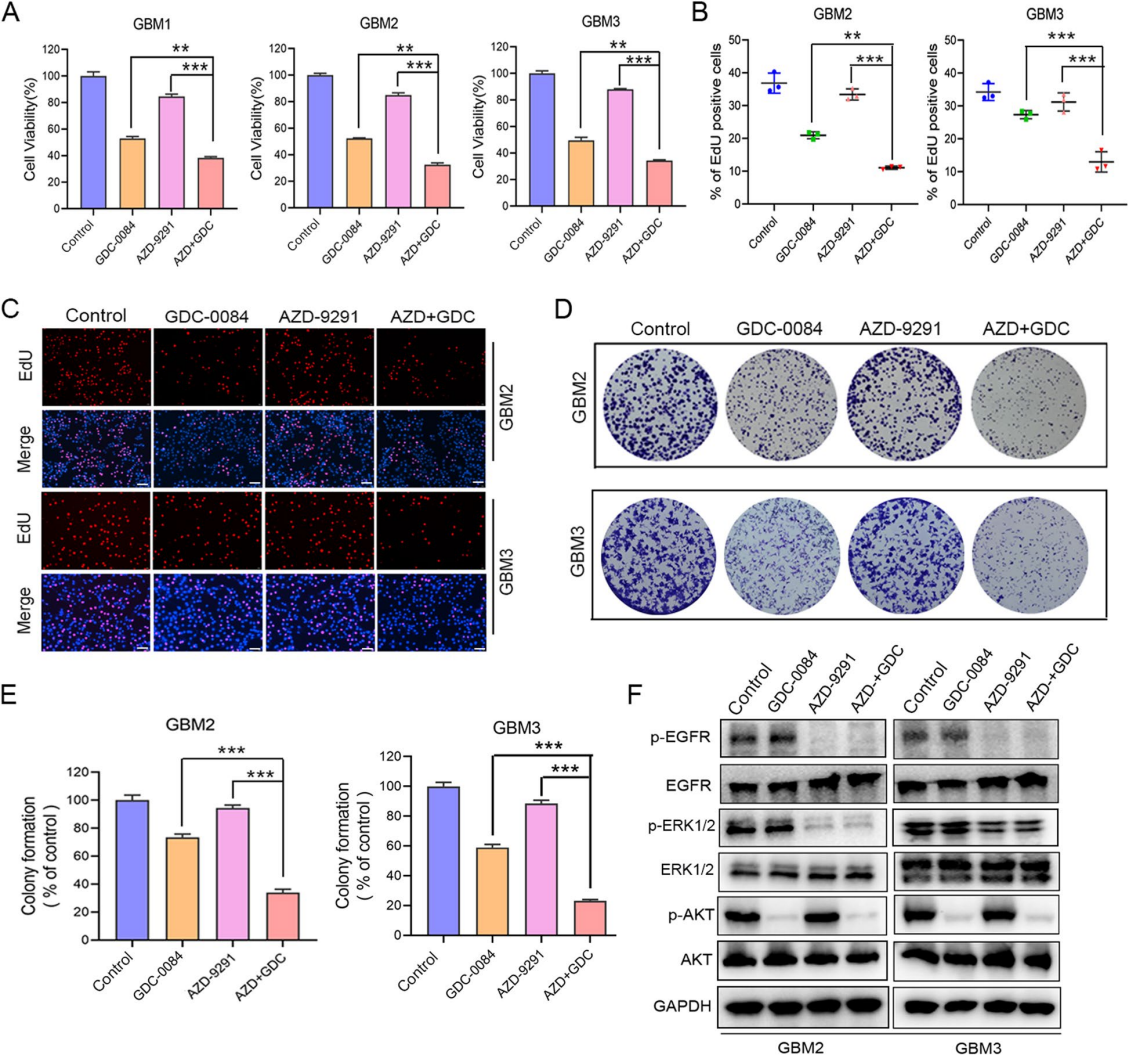
AZD-9291 combined with GDC-0084 synergistically decreases the survival and inhibits colony formation of primary GBM cells. **A** Three primary GBM cell lines were treated with AZD-9291 alone (1 μΜ), GDC-0084 alone (0.5 μΜ) or their combination for 72 h. Cell viability was then measured by CCK-8 assay. **B**, **C** Representative images of the EdU incorporation assay and Quantitative analysis of the results, scale bar: 100 μm. **D**, **E** Colony formation ability of GBM2 and GBM3 cells following AZD-9291 (1 μΜ) and/or GDC-0084 (0.5 μΜ). The numbers of colony formation were normalized to the control group. **F** GBM2 and GBM3 cells were exposed to 0.1%DMSO, 1 μΜ AZD-9291 alone, 0.5 μM GDC-0084 alone and AZD-9291 combined with GDC-0084 for 24 h. The expression levels of EGFR, p-EGFR, ERK1/2, p-ERK1/2, AKT and p-AKT were evaluated by Western blotting. GAPDH was used as loading control. All the Data are presented as means ± SD. **, *P* < 0.01, ***, *P* < 0.001

We performed CCK-8 assay to evaluate the antitumor activity of AZD-9291 and GDC-0084 combination therapy against primary cell lines derived from GBM tissues. The combination of the two drugs significantly inhibited the viability of the three primary cell lines (GBM1, GBM2 and GBM3) compared to AZD-9291 or GDC-0084 monotherapy (Fig. 4A). The CI values were < 1 for different concentrations of the treatment combination (Table S2, Supplementary information), indicating that AZD-9291 combined with GDC-0084 had synergistic inhibitory effects on the primary GBM cell lines.

EdU proliferation and colony formation assays were used to evaluate the therapeutic effect of the combination therapy on primary GBM cell proliferation. As shown in Figs. 4B–E, compared to the control group and the AZD-9291 or GDC-0084 monotherapy group, the percentage of EDU positive cells was significantly reduced in the AZD + GDC combination group to 11.06% in GBM2 cells and reduced to 12.99% in GBM3 cells, respectively (Fig. 4B, C). In addition, the number of colonies formed of GBM2 and GBM3 cells after the combination therapy was also significantly less than that in the control and monotherapy groups (Fig. 4D, E). Western blotting for GBM2 and GBM3 cells was performed to evaluate the effects of AZD-9291 and/or GDC-0084 treatment on the phosphorylation of key molecules (i.e., EGFR, ERK, and Akt) in the EGFR downstream signaling pathway. AZD-9291 monotherapy inhibited the levels of p-EGFR and p-ERK1/2, whereas GDC-0084 monotherapy inhibited the level of p-AKT. The combination of AZD-9291 and GDC-0084 simultaneously inhibited the phosphorylation levels of EGFR, ERK, and AKT (Fig. 4F). Our results further confirmed that AZD-9291 and GDC-0084 combination therapy simultaneously inhibited the MEK/ERK and AKT/mTOR signaling pathways downstream of EGFR, thereby inhibiting the growth of primary GBM cells.

### AZD-9291 combined GDC-0084 significantly inhibits the growth of subcutaneous and intracranial tumors and prolongs the survival of tumor-bearing mice

**Fig. 5:**
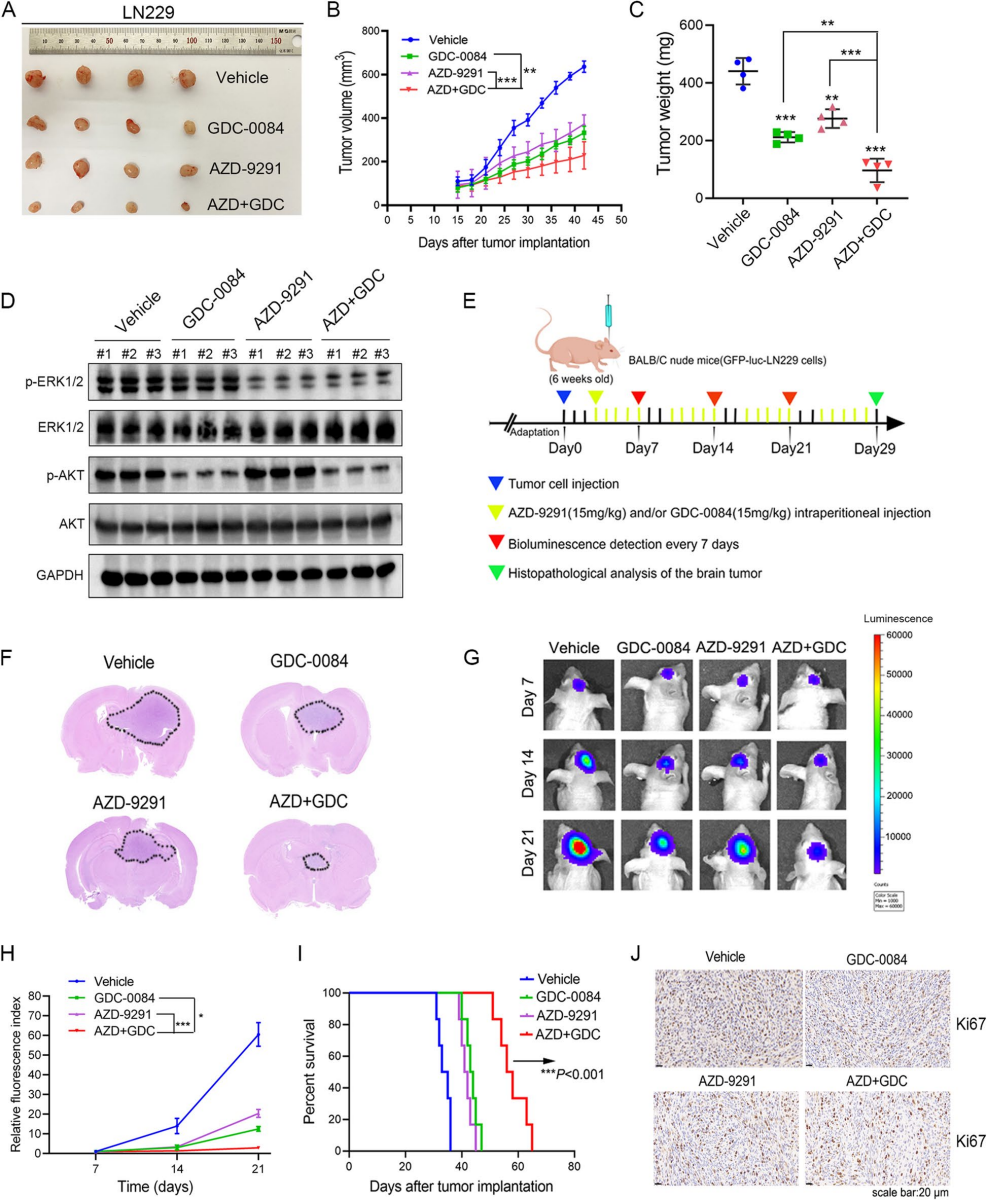
Combining AZD-9291 with GDC-0084 attenuated the growth of glioma in vivo. **A** Representative tumors isolated from the control, GDC-0084 (15 mg/kg), AZD-9291 (15 mg/kg) and AZD + GDC-treated groups of subcutaneous tumor model. **B** Tumor volume were recorded every 3 days. **C** Tumors isolated from each treatment group were weighted. The tumor weight was analyzed statistically. **D** Whole-tumor protein lysates were prepared from three randomly chosen tumors in each group to detect the levels of p-AKT and p-ERK1/2 using western blot analysis in vivo. **E** Schematic representation of the LN229-derived orthotopic xenograft experimental workflow. **F** Representative images of H&E staining of whole-brain sections from groups with different drugs administration. **G** Representative bioluminescence images of intracranial xenografts of each group on the indicated days after implantation. **H** Quantitative analysis of the results in (**G**). **I** Kaplan-Meier survival curves of mice implanted with LN229 cells with different drugs administration. A log-rank test was used to assess the statistical significance of the differences (*n* = 6). **J** Representative IHC staining images of Ki67 expression in LN-229-derived xenograft tumor of each group. Scale bar: 20 μm. * *P* < 0.05, ** *P* < 0.01, *** *P* < 0.001

AZD-9291 has been used in the clinical treatment of lung cancer. In addition, GDC-0084 is being evaluated in phase II clinical trials. In the present study, to verify the antitumor activity of the combination of the two drugs against GBM in vivo, we constructed a subcutaneous GBM tumor model in nude mice with LN229 cells and analyzed the volume and weight of tumor xenografts in different treatment groups. Our results showed that AZD-9291 or GDC-0084 monotherapy inhibited the growth of subcutaneous tumor cells in nude mice. However, the tumor volume of the mice in the AZD + GDC treatment group was significantly smaller than that of the control and monotherapy groups (Fig. 5A, B). The tumor weight of the AZD + GDC treatment group was also the smallest (Fig. 5C). We further verified the regulatory effects of AZD-9291 combined with GDC-0084 on the EGFR/ERK and AKT/mTOR signaling pathways in vivo. We extracted the total protein from tumor xenografts in each group and performed Western blotting. In vivo*,* GDC-0084 monotherapy only inhibited the activation of the AKT/mTOR signaling pathway, whereas AZD-9291 monotherapy only inhibited the ERK signaling pathway downstream of EGFR. However, the combination of AZD-9291 and GDC-0084 simultaneously inhibited the activation of the EGFR/ERK and AKT/mTOR signaling pathways, and more effectively inhibited GBM growth, compared to monotherapy (Fig. 5D). These results were consistent with the inhibitory effect of the combination therapy on the two signaling pathways in vitro.

Next, we evaluated the anti-GBM efficacy of the combination of AZD-9291 and GDC-0084 on orthotopic xenografts in mice. The LN229-derived orthotopic xenograft experimental workflow was shown in Fig. 5E. Hematoxylin and eosin staining showed that after AZD-9291 and GDC-0084 combination therapy, the intracranial tumors in mice grew slowly (Fig. 5F). The fluorescence results of small animal imaging revealed that the combination of AZD-9291 and GDC-0084 significantly inhibited the growth of intracranial tumors in mice (Fig. 5G, H). Survival analysis showed that the median survival time of tumor-bearing mice increased from 34 days in the control group to 57 days in the combination therapy group (Fig. 5I). The results of immunohistochemistry showed that the positive rate of Ki67 cells was significantly reduced after AZD-9291 and GDC-0084 combination therapy (Fig. 5J), further confirming that the combination therapy significantly inhibited the growth of GBM cells in vivo and prolonged the survival of tumor-bearing mice.

## Discussion

GBM is a grade 4 glioma with a poor prognosis and high recurrence rate. Due to the presence of the blood-brain barrier, many chemotherapeutic drugs fail to achieve effective therapeutic concentrations in the central nervous system. Hence, only few chemotherapeutic drugs can be used clinically for the treatment of GBM. Therefore, it is essential to develop new and effective targeted drugs or more effective treatment strategies for GBM. Our previous study showed that AZD-9291 had good antitumor activity on GBM cells. The present study evaluated the preclinical efficacy of dual inhibition of the EGFR/MEK/ERK and PI3K/AKT/mTOR signaling pathways for the treatment of GBM. The combination of AZD-9291 and GDC-0084 synergistically inhibited the proliferation of GBM cell lines and primary GBM cells by inducing cell cycle arrest. In addition, this combination therapy significantly slowed down the malignant progression of GBM cells in mice and prolonged the survival of tumor-bearing mice. Therefore, treatment combinations that simultaneously block the PI3K/AKT/mTOR and EGFR/MEK/ERK pathways may improve the therapeutic efficacy for GBM.

Abnormal activation of the PI3K/AKT/mTOR signaling pathway promotes the occurrence and progression of various cancers and plays a key role in the treatment of tumor drug resistance [19, 26]. The positive expression rate of PI3K/AKT/mTOR is closely related to the clinical grade of GBM and the poor prognosis of the patients [27, 28]. Thus, this pathway represents a potentially useful treatment target for GBM [29]. GDC-0084 is a dual-target inhibitor of PI3K and mTOR. Due to its physicochemical properties that allow brain penetration, this small molecule inhibitor was specifically developed as a potential drug treatment for GBM [21, 22]. GDC-0084 showed good antitumor activity in both GBM cell lines and glioma stem cells [30]. GDC-0084 induces cell cycle arrest in the G1 phase and inhibits Akt phosphorylation, thereby inhibiting tumor progression [22, 31, 32]. In this study, GDC-0084 alone had significant inhibitory effect against various GBM cell lines and primary GBM cells, by blocking the cell cycle progression, inhibiting the phosphorylation of AKT, mTOR, p70S6, and 4E-BP1, and subsequently blocking the PI3K/AKT/mTOR signaling pathway to inhibit GBM growth. Nevertheless, GDC-0084 monotherapy had no significant effect on the ERK pathway. In vivo experiments further verified the antitumor efficacy of GDC-0084 in GBM. The results of completed and ongoing clinical trials in GBM patients have shown that GDC-0084 has good pharmacokinetic properties and an acceptable safety profile with positive pharmacodynamic effects in GBM. In particular, 40% of GBM patients achieved stable disease and 55% developed progressive disease [23]. Thus, GDC-0084 may be a potential drug target for GBM.

Multiple signaling pathways are simultaneously activated in GBM, including the EGFR, PI3K/Akt, and MEK/ERK pathways. EGFR, as an upstream key regulatory molecule of the two signaling pathways of PI3K/AKT and MEK/ERK, plays a critical role in carcinogenesis in GBM occurrence and progression [33, 34]. Our previous study has shown that the third-generation EGFR inhibitor AZD-9291 has good anti-tumor activity on GBM cells in vitro and in vivo [15]. In addition, AZD-9291 is better tolerated than the first- and second-generation EGFR-tyrosine kinase inhibitors [35]. Unfortunately, no completed clinical trials of AZD-9291 have included patients with GBM. Some studies have evaluated the therapeutic efficacy of AZD-9291 in GBM [16, 36]. Abousaud et al. reported the effectiveness of AZD-9291 in recurrent GBM with EGFR alterations. Among the four GBM patients who underwent evaluation for the response after AZD-9291 treatment, one achieved partial response, two achieved stable disease while taking AZD-9291, and one exhibited no response [16]. The results provide preliminary evidence that AZD-9291 has a tolerable safety profile in patients with brain tumors and may benefit patients with recurrent GBM and EGFR alterations. Given its good permeability of the blood-brain barrier, AZD-9291 may be effective in prolonging the survival of a subset of patients with EGFR-mutant GBM. It is difficult to achieve cure with monotherapy. Thus, it is essential to explore additional combination therapies for the treatment of GBM.

The PI3K/Akt and the MAPK/ERK pathways have extensive cross-talk in regulating cell survival. Inhibition of one of the pathways leads to compensatory activation of the other pathway [37, 38]. Therefore, AZD-9291 combined with GDC-0084 may be particularly effective for the treatment of GBM. In present study, AZD-9291 or GDC-0084 (inhibitor) alone was not effective in preventing cell growth or prolonging survival when concentrations below 2 μM were used. However, the cell viability was significantly reduced after the combination therapy (Fig. 1A–D). Furthermore, the CI was < 1 in vitro assay, which indicated that the combination of AZD-9291 and GDC-0084 had a synergistic inhibitory effect. In addition, the volume of subcutaneous and intracranial tumors after AZD + GDC combined treatment was significantly smaller than that after AZD-9291 or GDC-0084 monotherapy. The median survival of mice was also significantly prolonged after combined treatment. Although the PI3K/Akt pathway is known to be downstream of EGFR activation [39, 40], treatment of AZD-9291 alone has little or no effect on PI3K/Akt activity. This is consistent with the findings of other EGFR inhibitors such as gefitinib and erlotinib [39, 41]. Previous study has found that in cells resistant to EGFR inhibitors, the PI3K/AKT signaling pathway may be activated by other upstream kinases such as MET and IGF1R [42]. Overexpression of MET has been found in tissues derived from glioma patients. Targeted inhibition of the MET/PI3K/AKT signaling pathway enhances the sensitivity of lung cancer cells to EGFR inhibitors [43]. However, further investigation is needed to evaluate the role of MET in intrinsic resistance to EGFR inhibitors and differential response to PI3K/Akt inhibitors and EGFR inhibitors combination in GBM cell lines. Mechanistically, we found that the combination of GDC-0084 and AZD-9291 simultaneously blocked the activation of PI3K/AKT and EGFR/MEK/ERK signaling, significantly improving the antitumor activity in vitro and in vivo. Hence, simultaneous activation of multiple signaling pathways may be the key driving force for GBM cell proliferation and survival. To achieve maximal antitumor activity, targeted combination therapies may be required to simultaneously block multiple survival pathways.

## Conclusions

Taken together, our results demonstrated that AZD-9291 combined with GDC-0084 synergistically inhibited the proliferation and colony survival of GBM and primary GBM cells. Furthermore, it significantly slowed the tumor growth in preclinical orthotopic models. The AZD-9291 and GDC-0084 combination therapy inhibited the proliferation of GBM cells by simultaneously blocking the EGFR/ERK and PI3K/AKT/mTOR signaling pathways, thereby blocking cell cycle progression. Therefore, multiple signaling nodes co-targeting the EGFR/MEK/ERK and the PI3K/AKT/mTOR pathways may have potential therapeutic effects. This study provided a promising therapeutic strategy for the treatment of GBM.

### Supplementary Information

**Additional file 1: Table S1.** Combination index (CI) for the synergistic combination of GDC-0084 and AZD-9291 in LN229, U251, U87 and T98G cells. **Table S2.** Combination index (CI) for the synergistic combination of GDC-0084 and AZD-9291 in primary GBM cells. **Figure S1.** Combinatorial treatment with AZD-9291 and GDC-0084 significantly inhibits the proliferation of T98G and U87 cells. **Figure S2.** Combinatorial treatment with AZD-9291 and GDC-0084 significantly inhibits colony formation in U87 and T98G cells. **Figure S3.** AZD-9291 and GDC-0084 combination induces cell cycle arrest.

## Data Availability

The datasets supporting the conclusions of this article are included within the article.

## Abbreviations

GBM: Glioblastoma multiforme
EGFR: Epidermal growth factor receptor
PI3K: Phosphoinositide 3-kinase
mTOR: Mammalian target of rapamycin
CCK-8: Cell counting kit-8
DAPI: 4′,6-diamidino-2-phenylindole
EdU: 5-ethynyl-2′-deoxyuridine
KEGG: Kyoto Encyclopedia of Genes and Genomes
GSEA: Gene set enrichment analysis
