# Supplementary material for: Dual blockade of EGFR and PI3K signaling pathways offers a therapeutic strategy for glioblastoma
Source: Cell Commun Signal. 2023 Dec 18;21:363. doi: 10.1186/s12964-023-01400-0 (PMC10729576; doi:10.1186/s12964-023-01400-0)
Supplement: Supplementary file 2 — Additional file 1: Table S1. Combination index (CI) for the synergistic combination of GDC-0084 and AZD-9291 in LN229, U251, U87 and T98G cells. Table S2. Combination index (CI) for the synergistic combination of GDC-0084 and AZD-9291 in primary GBM cells. Figure S1. Combinatorial treatment with AZD-9291 and GDC-0084 significantly inhibits the proliferation of T98G and U87 cells. Figure S2. Combinatorial treatment with AZD-9291 and GDC-0084 significantly inhibits colony formation in U87 and T98G cells. Figure S3. AZD-9291 and GDC-0084 combination induces cell cycle arrest. [file 12964_2023_1400_MOESM1_ESM.docx]

**Supplementary information**

**Table S1. Combination index (CI) for the synergistic combination of GDC-0084 and AZD-9291 in LN229, U251, U87 and T98G cells**

| GDC-0084  (μΜ) | AZD-9291  (μΜ) | LN229 | | U251 | | U87 | | T98G | |
| --- | --- | --- | --- | --- | --- | --- | --- | --- | --- |
|  |  | **Inhibition Ratio** | **CI Value*** | **Inhibition Ratio** | **CI Value*** | **Inhibition Ratio** | **CI Value*** | **Inhibition Ratio** | **CI Value*** |
| 0.3125 | 0.3125 | 0.390 | 0.60579 | 0.199 | 0.84167 | 0.213 | 0.90390 | 0.144 | 1.31273 |
| 0.625 | 0.625 | 0.616 | 0.56582 | 0.331 | 0.96769 | 0.402 | 0.88871 | 0.324 | 1.10098 |
| 1.25 | 1.25 | 0.750 | 0.67462 | 0.587 | 0.83208 | 0.607 | 0.92730 | 0.613 | 0.81376 |
| 2.5 | 2.5 | 0.816 | 0.97861 | 0.742 | 0.94649 | 0.837 | 0.72897 | 0.798 | 0.76318 |
| 5.0 | 5.0 | 0.915 | 0.94526 | 0.903 | 0.74389 | 0.93 | 0.70101 | 0.895 | 0.80647 |
| 10 | 10 | 0.982 | 0.49312 | 0.968 | 0.58511 | 0.982 | 0.46122 | 0.981 | 0.36710 |

*CI value less than 1 indicated that there is a synergistic effect of GDC-0084 combined with AZD-9291.

**Table S2. Combination index (CI) for the synergistic combination of GDC-0084 and AZD-9291 in primary GBM cells**

| GDC-0084  (μΜ) | AZD-9291  (μΜ) | GBM1 | | GBM2 | | GBM3 | |
| --- | --- | --- | --- | --- | --- | --- | --- |
|  |  | **Inhibition Ratio** | **CI Value*** | **Inhibition Ratio** | **CI Value*** | **Inhibition Ratio** | **CI Value*** |
| 0.25 | 0.5 | 0.498 | 0.41973 | 0.438 | 0.67031 | 0.479 | 0.60242 |
| 0.25 | 1.0 | 0.517 | 0.37676 | 0.529 | 0.43482 | 0.489 | 0.61206 |
| 0.5 | 0.5 | 0.576 | 0.47920 | 0.578 | 0.59195 | 0.631 | 0.55836 |
| 0.5 | 1.0 | 0.616 | 0.36253 | 0.674 | 0.34774 | 0.656 | 0.50584 |

*CI value less than 1 indicated that there is a synergistic effect of GDC-0084 combined with AZD-9291.


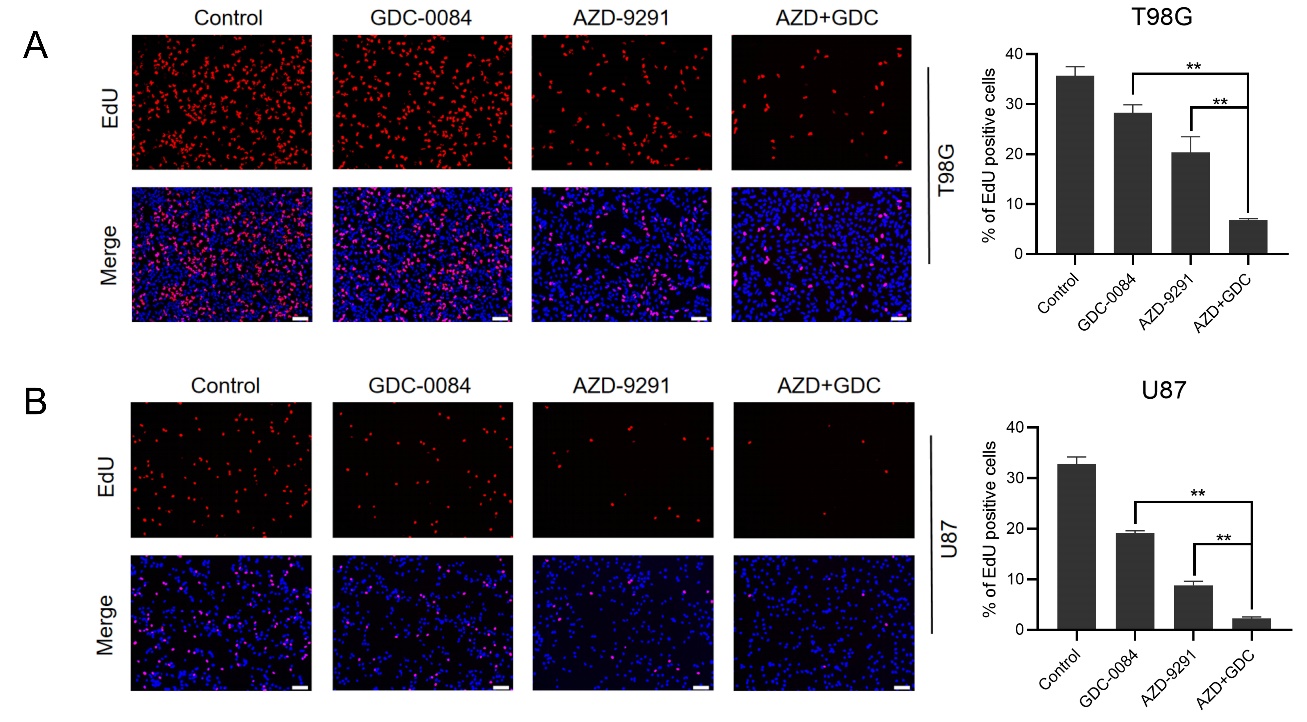


**Figure.S1 Combinatorial treatment with AZD-9291 and GDC-0084 significantly inhibits the proliferation of T98G and U87 cells**. (A and B) T98G and U87 cells were treated with AZD-9291 (2 μΜ) and/or GDC-0084 (2 μΜ) for 24 h. Cell proliferation was evaluated by EdU incorporation assay. Quantitative results were analyzed. All the data were presented as means ± SD from three independent experiments (***P* < 0.01), *scale bar:* 100 μm.


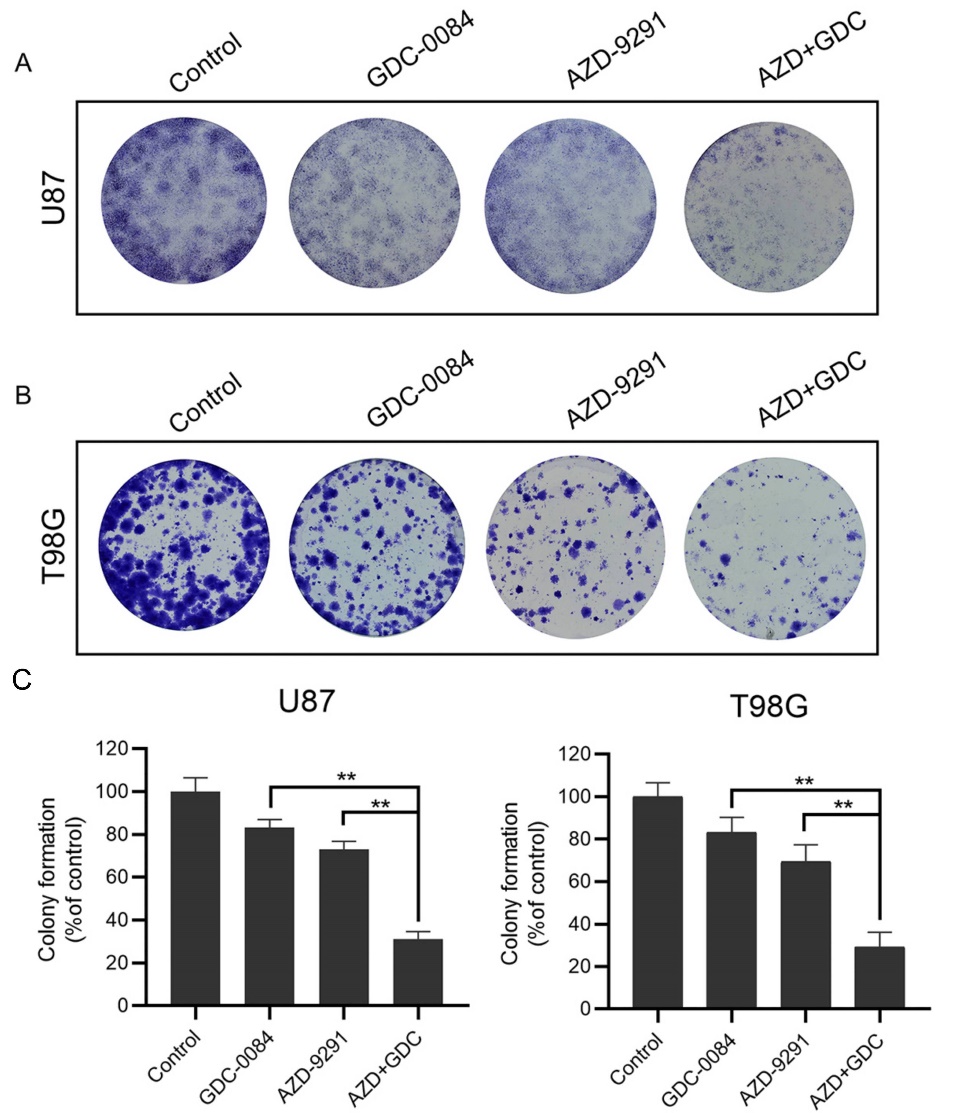


**Figure.S2 Combinatorial treatment with AZD-9291 and GDC-0084 significantly inhibits colony formation in U87 and T98G cells**. (A and B) U87 and T98G cells were treated with AZD-9291 (2 μΜ) and/or GDC-0084 (2 μΜ) for 24 h, and then changed with drug-free medium for another 14 days. The numbers of colony formation were counted. Quantitative analysis of the results was shown in (C). The numbers of colony formation were normalized to the control group. All the data were presented as means ± SD from three independent experiments (***P* < 0.01).


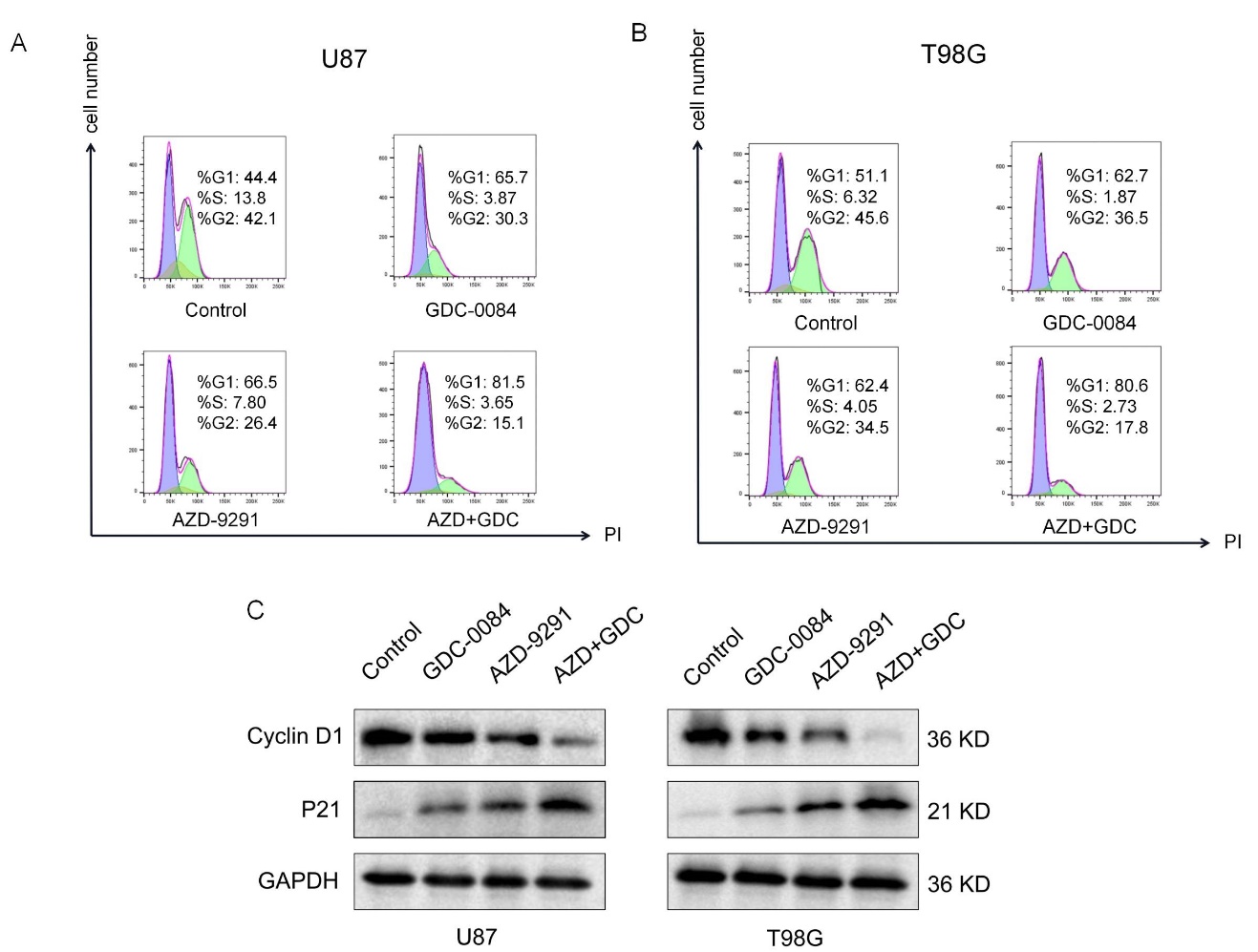


**Figure.S3 AZD-9291 and GDC-0084 combination induces cell cycle arrest**. (A and B) After treatment with AZD-9291 (2 μΜ) and/or GDC-0084 (2 μΜ) for 24 h, the cell cycle was measured by flow cytometry in U87 and T98G cells. (C) U87 and T98G cells were treated with AZD-9291 (2 μΜ) and/or GDC-0084 (2 μΜ) for 24 h. Cell lysates were analyzed for Cyclin D1 and p21 expression by western blot assay.
